# Racial and Ethnic Differences in Liver Transplantation and Post–Liver Transplant Survival Among Patients With Hepatocellular Carcinoma

**DOI:** 10.1002/cam4.70298

**Published:** 2024-11-06

**Authors:** Udhayvir S. Grewal, Apoorva K. Chandar, Shiva J. Gaddam, Abdul Rahman Al Armashi, Akram Alkreshi, Subhash C. Garikipati

**Affiliations:** ^1^ University of Iowa Hospitals and Clinics Iowa City Iowa USA; ^2^ University Hospitals Cleveland Medical Center Cleveland Ohio USA; ^3^ Louisiana State University Health Sciences Center Shreveport Louisiana USA; ^4^ MetroHealth Medical Center Cleveland Ohio USA; ^5^ Virginia Tech Carillion School of Medicine Roanoke Virginia USA

## Abstract

**Background:**

Racial and ethnic disparities in diagnosis and overall outcomes for HCC are well known. We present updated real‐world data on racial or ethnic differences in LT and post‐LT survival among patients with HCC in a large population‐based database.

**Methods:**

We used the TriNetX database to retrospectively identify patients who had HCC (ICD‐10 C22.0, C22.8) and underwent LT (CPT codes 47,135, 47,140, 47,140, 47,141, 47,142) from 2012 to 2022 and compared outcomes across racial and ethnic subgroups.

**Results:**

Majority of the patients were Caucasians (2403/2901, 84.8%), followed by African Americans (267/2901, 9.2%) Hispanic/Asian (231/2901, 7.9%). At follow up of 5 years, we noted no significant difference in mortality between AA and Caucasian patients [HR = 1.087 (95% CI 0.76, 1.56, *p* = 0.59)] as well as Hispanic/Asian and Caucasian patients [HR = 1.14 (95% CI 0.73, 1.78, *p* = 0.10)].

**Conclusions:**

These results indicate that stringent implementation of policies aimed at ensuring equitable access to LT may contribute to bridging disparities in overall outcomes in HCC.

## 1 Introduction

Hepatocellular carcinoma (HCC) is the most common type of primary liver cancer and is also a leading cause of mortality among patients with chronic liver disease and cirrhosis [1]. The rates of mortality related to HCC have been shown to be declining in the United States (US) due to recent efforts geared towards early detection and advancements in therapeutic options; especially curative surgical treatments (hepatic resection and transplant) [2]. Despite these efforts, racial and ethnic disparities in HCC‐related outcomes continue to persist. Racial and ethnic minorities with HCC in the US have consistently been shown to have relatively poorer overall survival, especially due to lower rates of curative HCC treatment [3].

Liver transplantation (LT) is a potentially curative treatment option for some patients with early‐stage hepatocellular carcinoma or HCC (based on Milan criteria) [4]. However historically, racial and ethical minorties have been shown to be less likely to be referred for LT and also subsequently undergo LT [5, 6]. The implementation of newer policies, such as Share 35, seeks to address these key disparities in organ allocation and improve access to LT across racial and ethnic subgroups [7]. We present updated real‐world data on racial or ethnic differences in LT and post‐LT survival among patients with HCC in a large population‐based database.

## 2 Materials and Methods

We used TriNetX, a global federated research network maintaining and providing statistics on electronic health record (EHR)‐based patient data. For the current analysis, we extracted EHR data from 74 million patients across 54 healthcare organizations in the US. Since the study did not involve access or reporting of protected health information, institutional ethics review board approval was not sought. The study was conducted in accordance with the Declaration of Helsinki.

We retrospectively identified patients who had an ICD‐10 diagnosis of HCC (ICD‐10 codes C22.0, C22.8) and had undergone LT (CPT codes 47,135, 47,140, 47,140, 47,141, 47,142) from 2012 to 2022. We retrieved data on baseline characteristics such as age, race, sex, comorbidities, etc. and compared them across racial and ethnic subgroups; Caucasians, African Americans (AA), and other minorities (Hispanics/Asians) using the Chi‐square test.

We then studied post‐LT outcomes using survival at 1, 3, and 5 years using propensity‐matched cohorts across various racial sub‐groups. Propensity score matching was performed 1:1 using age, sex and selected co‐morbidities. In‐built statistical tools were used to perform statistical analysis; Chi‐square test was used for comparisons between groups. Kaplan–Meier survival estimates were plotted for long‐term mortality analysis at 5‐year follow up of the cohort. A *p* < 0.05 was considered statistically significant.

## 3 Results

A total of 2901 patients with HCC underwent LT over the study period, out of which, the majority were Caucasians (2403/2901, 84.8%), followed by AA (267/2901, 9.2%) Hispanic/Asian (231/2901, 7.9%).

At baseline, AA patients had a higher prevalence of viral hepatitis B (10.1% vs. 5.7%, *p* = 0.004), hepatitis C (72.7% vs. 47.1%, *p* < 0.001), and lower prevalence of alcoholic liver disease (36.3% vs. 45.5%, *p* = 0.004) as compared to White patients. Hispanic/Asian patients had a lower prevalence of alcoholic liver disease (37.7% vs. 45.5%, *p* = 0.02), nicotine dependence (13.4% vs. 21.3%, *p* = 0.004) and higher prevalence of viral hepatitis B (24.7% vs. 5.7%, *p* < 0.001) as compared to Caucasian patients. Baseline characteristics before and after propensity score matching are shown in Tables 1 and 2 respectively.

**TABLE 1:** Baseline characteristics of patients included in the study cohort across racio‐ethnic study groups before propensity score matching.

Parameters	Caucasian patients (*N* = 2403)	AA patients (*N* = 267)	*p* ^a^	Hispanic/Asian patients (*N* = 231)	*p* ^b^
Age (years)	60.4 ± 7.8	59.7 ± 8.5	0.17	60.3 ± 7.8	0.91
Female sex	23.4	24.2%	0.77	20.8%	0.36
Mean BMI (kg/m^2^)	28.8 ± 6.0	29.5 ± 5.6	0.92	27.8 ± 5.2	0.01
Alcoholic liver disease	45.6%	36.3%	0.003	37.7%	0.02
Hepatitis B	5.6%	10.3%	0.002	24.7%	< 0.01
Hepatitis C	47.1%	72.2%	< 0.001	42.9%	0.21
Nicotine dependence	21.3%	26.4%	0.05	13.4%	0.004
Diabetes mellitus	49.8%	49.5%	0.92	48.9%	0.82
Hypertension	68.2%	81.7%	< 0.01	66.7%	0.63
Chronic kidney disease	26.7%	32.6%	0.04	22.9%	0.20
COPD	9.6%	13.2%	0.06	4.8%	0.01

Abbreviations: AA, African American; BMI, Basal Metabolic Index; COPD, chronic obstructive pulmonary disease.

^a^ *p*‐value between White and AA patients.

^b^ *p*‐value between White and Hispanic/Asian patients.

**TABLE 2:** Baseline characteristics of patients included in the study cohort across racio‐ethnic study groups after propensity score matching.

Parameters	Caucasian patients (*N* = 265)	AA patients (*N* = 265)	*p* ^a^	White patients (*N* = 220)	Hispanic/Asian patients (*N* = 220)	*p* ^b^
Age (years)	59.9 ± 6.8	59.7 ± 8.6	0.74	60.8 ± 7.9	60.5 ± 7.8	0.72
Female sex	20.8%	24.5%	0.29	21.4%	21.8%	0.91
Mean BMI (kg/m^2^)	28.8 ± 6.0	29.5 ± 5.8	0.92	28.6 ± 5.0	27.8 ± 5.2	0.34
Alcoholic liver disease	41.5%	36.2%	0.21	42.3%	39.5%	0.56
Hepatitis B	8.3%	9.4%	0.65	20.9%	20.9%	1.00
Hepatitis C	75.5%	72.5%	0.43	39.5%	44.5%	0.29
Nicotine dependence	25.3%	26.0%	0.84	15.9%	14.1%	0.59
Diabetes mellitus	52.1%	49.8%	0.60	54.5%	45.9%	0.29
Hypertension	83.8%	81.5%	0.49	66.8%	68.2%	0.76
Chronic kidney disease	27.5%	32.5%	0.22	25%	23.6%	0.74
COPD	11.7%	13.2%	0.59	4.5%	5.0%	0.82

Abbrevaitions: AA, African Americans; BMI, Basal Metabolic Index; COPD, chronic obstructive pulmonary disease.

^a^ *p*‐value between White and AA patients.

^b^ *p*‐value between White and Hispanic/Asian patients.

**FIGURE 1:**
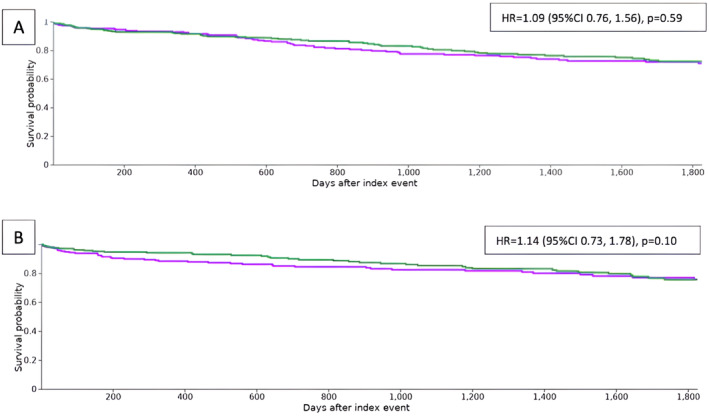
Kaplan–Meier survival estimates at 5 years between (A) African American and Caucasian patients (Caucasian patients represented by the green curve) and (B) Hispanic/Asian and Caucasian patients (Caucasian patients represented by the green curve). CI, confidence intervals; HR, hazard ratio.

In propensity‐matched cohorts, we found no differences post‐LT survival at 1 year (92.9% vs. 91.6%, *p* = 0.60), 3 years (77.6% vs. 80.5%, *p* = 0.48), and 5 years (71.0% vs. 72.3%, *p* = 0.46) between AA and Caucasian patients. While post‐LT survival at 1 year was lower among Hispanic/Asian patients (88.5% vs. 94.3%, *p* = 0.04), we found no difference in post‐LT survival at 3 years (82.5% vs. 85.4%, *p* = 0.27) and 5 years (75.8% vs. 75.7%, *p* = 0.55) when compared to Caucasians. Overall, at follow up of 5 years (Figure 1), we noted no significant difference in mortality between AA and Caucasian patients [HR = 1.087 (95% CI 0.76,1.56, *p* = 0.59)] as well as Hispanic/Asian and Caucasian patients [HR = 1.14 (95% CI 0.73, 1.78, *p* = 0.10)].

## 4 Discussion

We present real‐world data demonstrating the under‐representation of racial minorities in this U.S. cohort of patients with HCC receiving LT. Our findings highlight persistent disparities in the rates of LT among racial and ethnic minorities with HCC. Among patients who underwent LT, other than higher early mortality among Hispanic/Asian subgroup, we did not detect any differences in long‐term post‐LT survival between racial and ethnic subgroups.

Racial distribution for LT in our analysis appears to be substantially disproportionate to the distribution of racial/ethnic minorities in early‐stage HCC in the U.S. [8] Recent data continue to show that Black patients are significantly less likely to undergo LT for HCC compared to Whites [9, 10]. Similarly, other studies indicate a lower rate of LT among Hispanic and Asian patients with HCC when compared to Whites [11]. These data allude to key disparities in organ allocation that continue to persist despite key policy changes [7].

In the current analysis, we found no differences in post‐LT survival (except higher early mortality among Hispanic/Asian patients), across racial/ethnic groups. Existing data regarding post‐LT survival among patients with HCC are conflicting. A retrospective analysis from the United Network Organ Sharing/Organ Procurement Transplant Network showed that Blacks with HCC had worse post‐LT compared to other races [12]. However, other studies have demonstrated a significant improvement in post‐LT survival due to the increasing availability of direct‐acting antiviral therapy for hepatitis C infection (a major risk factor for HCC in Black patients) [13].

The current study has limitations. Apart from being limited by its retrospective design, the analysis was performed using a database which does not allow access to granular patient‐level data which limits the ability to adjust for potential confounders and provide a more detailed analysis to further enrich existing body of evidence in this area. However, these limitations are inherent to most large multi‐institutional databases of this nature. Additionally, the current analysis is also limited by the abiliyt of TriNetX to capture all patients who underwent LT for HCC in the U.S.

Despite these limitations, our study offers important data that underscore the need for stringent implementation of policies aimed at ensuring equitable access to LT among patients with HCC. Since our study did not demonstrate any race‐based differenes in long‐term post‐LT survival, we hypothesize that focused efforts aimed at improved access to LT may contribute towards bridging of racial disparities in overall outcomes in HCC.

## Author Contributions

**Udhayvir S. Grewal:** conceptualization (lead), formal analysis (equal), investigation (lead), methodology (lead), project administration (lead), writing – original draft (lead), writing – review and editing (equal). **Apoorva K. Chandar:** data curation (equal), investigation (equal), methodology (equal), writing – review and editing (equal). **Shiva J. Gaddam:** investigation (equal), writing – original draft (equal), writing – review and editing (equal). **Abdul Rahman Al Armashi:** writing – original draft (equal), writing – review and editing (equal). **Akram Alkreshi:** writing – original draft (equal), writing – review and editing (equal). **Subhash C. Garikipati:** writing – original draft (equal), writing – review and editing (equal).

## Conflicts of Interest

The authors declare no conflicts of interest.

## Data Availability

The authors have nothing to report.
